# Supplementary material for: Glycoproteomics-Compatible MS/MS-Based Quantification of Glycopeptide Isomers
Source: Anal Chem. 2023 Jun 15;95(25):9605–14. doi: 10.1021/acs.analchem.3c01319 (PMC10308332; doi:10.1021/acs.analchem.3c01319)
Supplement: Supplementary file 1 — ac3c01319_si_001.pdf [file ac3c01319_si_001.pdf]

Supplementary information

Glycoproteomics-compatible MS/MS-based quantification of glycopeptide isomers

Authors: Joshua C.L. Maliepaard[1,2], J. Mirjam A. Damen[1,2], Geert-Jan P.H. Boons[3,4,5], Karli R. Reiding[1,2,*]

[1]*Biomolecular Mass Spectrometry and Proteomics, Utrecht Institute for Pharmaceutical Sciences and Bijvoet Center for Biomolecular Research, University of Utrecht, Utrecht, 3584 CH, the Netherlands;*

[2]*Netherlands Proteomics Center, Utrecht, 3584 CH, the Netherlands;*

[3]*Department of Chemical Biology and Drug Discovery, Utrecht Institute for Pharmaceutical Sciences and Bijvoet Center for Biomolecular Research, University of Utrecht, Utrecht, 3584 CG, the Netherlands;*

[4]*Complex Carbohydrate Research Center, University of Georgia, Athens, GA 30602, USA;*

[5]*Department of Biochemistry and Molecular Biology, University of Georgia, Athens, GA 30602, USA;*

*Correspondence may be addressed to: k.r.reiding@uu.nl

Table of contents

Figure S1	S3
Figure S2	S4
Figure S3	S5
Figure S4	S6
Figure S5	S7
Figure S6	S8
Figure S7	S9
Figure S8	S10
Figure S9	S11
Table S1	S12
Table S2	S13
Table S3	S14
Supplementary Information 1	S15-S25
Supplementary Information 2	S26-S32

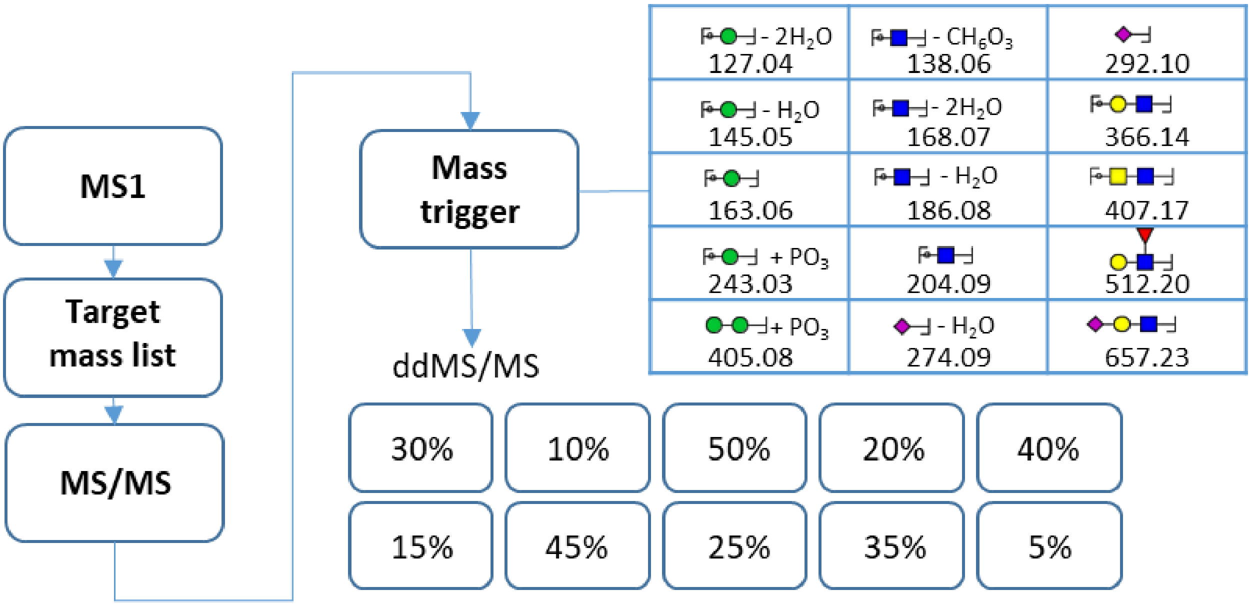

Figure S1. Stepped HCD LC-MS/MS method employed throughout the experiments. Following MS1, the LC-MS/MS method selected precursors from a target mass list, covering, depending on the application, isomeric standards, sialylglycopeptides and trastuzumab glycopeptides with 2+ charge. Hereafter an MS/MS prescan was performed at HCD NCE 29%. If this prescan contained at least 3 detections matching a mass trigger list with glycan-specific oxonium ions, a series of stepped HCD MS/MS scans was triggered covering NCEs 5-50% in steps of 5%, following a semi-randomized fashion. The maximum time between MS1 scans was set to 3 seconds. Since our chromatography provided us with peaks that were usually more than 30 seconds with, this cycle time allowed for 10 data points per peak. The target mass list was used to maximize the selection of our compounds of interest, but can be removed or replaced with for example a *m/z* range.

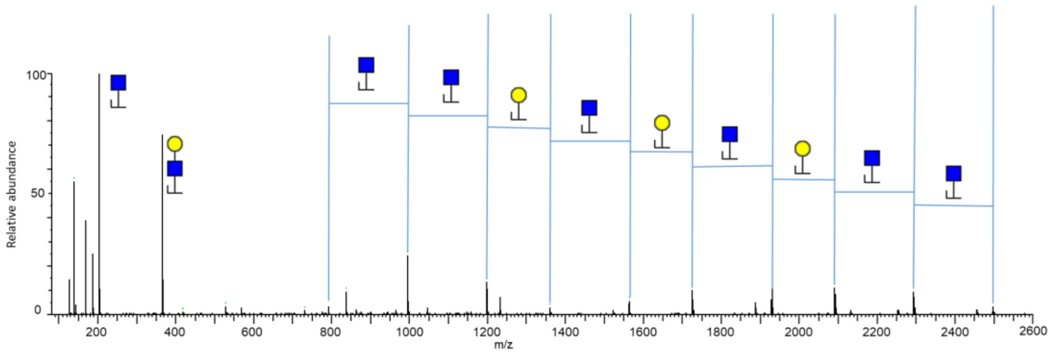

Figure S2: MS/MS spectrum showing an incorrect fragmentation pattern for SGP KVANKT-H4N5S2. While having the same *m/z* value as KVANKT-H4N5S2 (*m/z* 1433.0890 at z=2+), the spectrum above clearly displays at least 6 HexNAc residues, suggesting a different glycan entirely. To avoid false assignments of glycopeptides such as these, an intensity threshold for certain oxonium ions was implemented, in this case a minimum of $1 \cdot 10^4$ for *m/z* 204.0867, 366.1395 and 657.2349 and $5 \cdot 10^3$ for *m/z* 274.0921 and 292.1027.

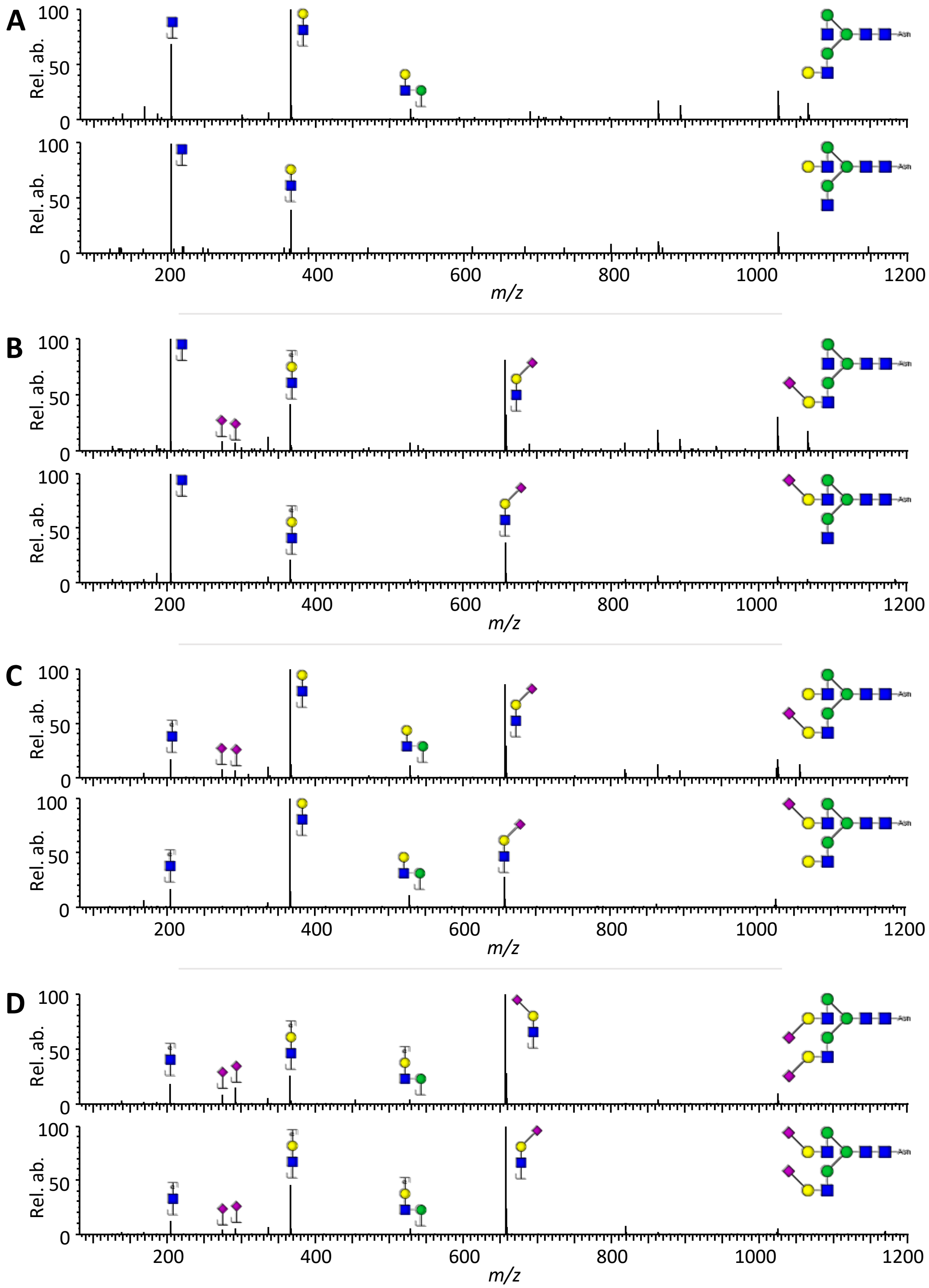

Figure S3: MS/MS spectra of glycosylated asparagine standards at 15% NCE A) N4H4 glycosylated asparagines show higher relative abundance of HexHexNAc (*m/z* 366.1395) when the galactose is positioned on the 3-branch compared to the 6-branch. **B)** N4H4S1 glycosylated asparagines show higher relative abundance of HexHexNAc (*m/z* 366.1395) and NeuAcHexHexNAc (*m/z* 657.2349) when the sialylated galactose is positioned on the 3-branch compared to the 6-branch. **C)** N4H5S1 glycosylated asparagines show higher relative abundance of NeuAcHexHexNAc (*m/z* 657.2349) when the sialic acid is positioned on the 3-branch compared to the 6-branch. **D)** N4H5S2 glycosylated asparagines show higher relative abundance of NeuAc-H2O (*m/z* 274.0921) and NeuAc (*m/z* 292.1027) when the sialic acids are α2,3-linked compared to α2,6-linked.

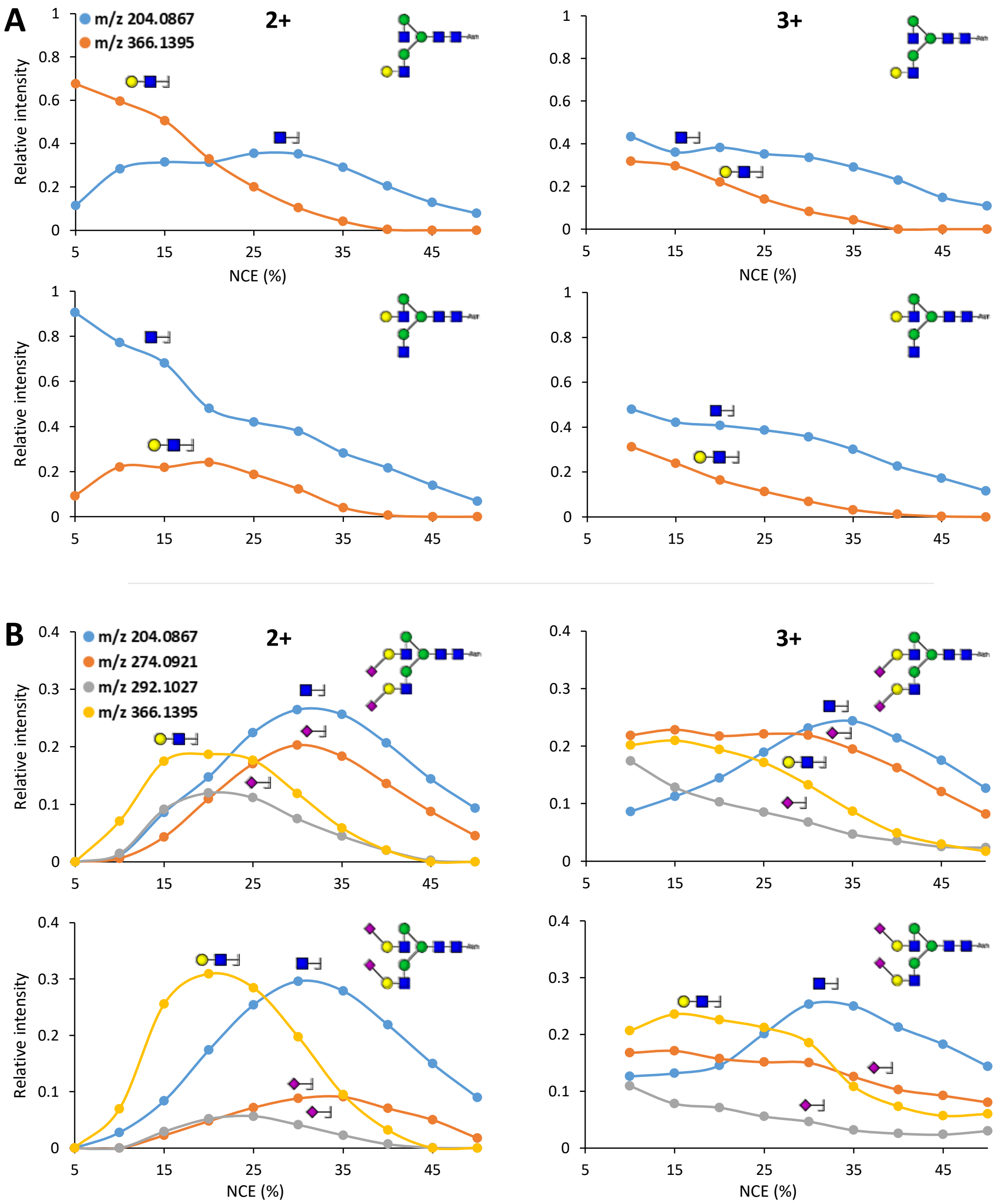

Figure S4: MS/MS fragmentation changes for charge states 2+ and 3+. **A**) Relative intensity differences between the oxonium ions for HexNAc and HexHexNAc, compared between galactose isomers, for precursor charge states 2+ (**left**) and 3+ (**right**). **B**) Relative intensity differences between the oxonium ions for HexNAc, HexHexNAc, NeuAc-H_2O and NeuAc, compared between sialic acid linkage isomers, for precursor charge states 2+ (**left**) and 3+ (**right**). As can be seen, the ion ratio differences between isomers proved more pronounced in 2+ when compared to 3+.

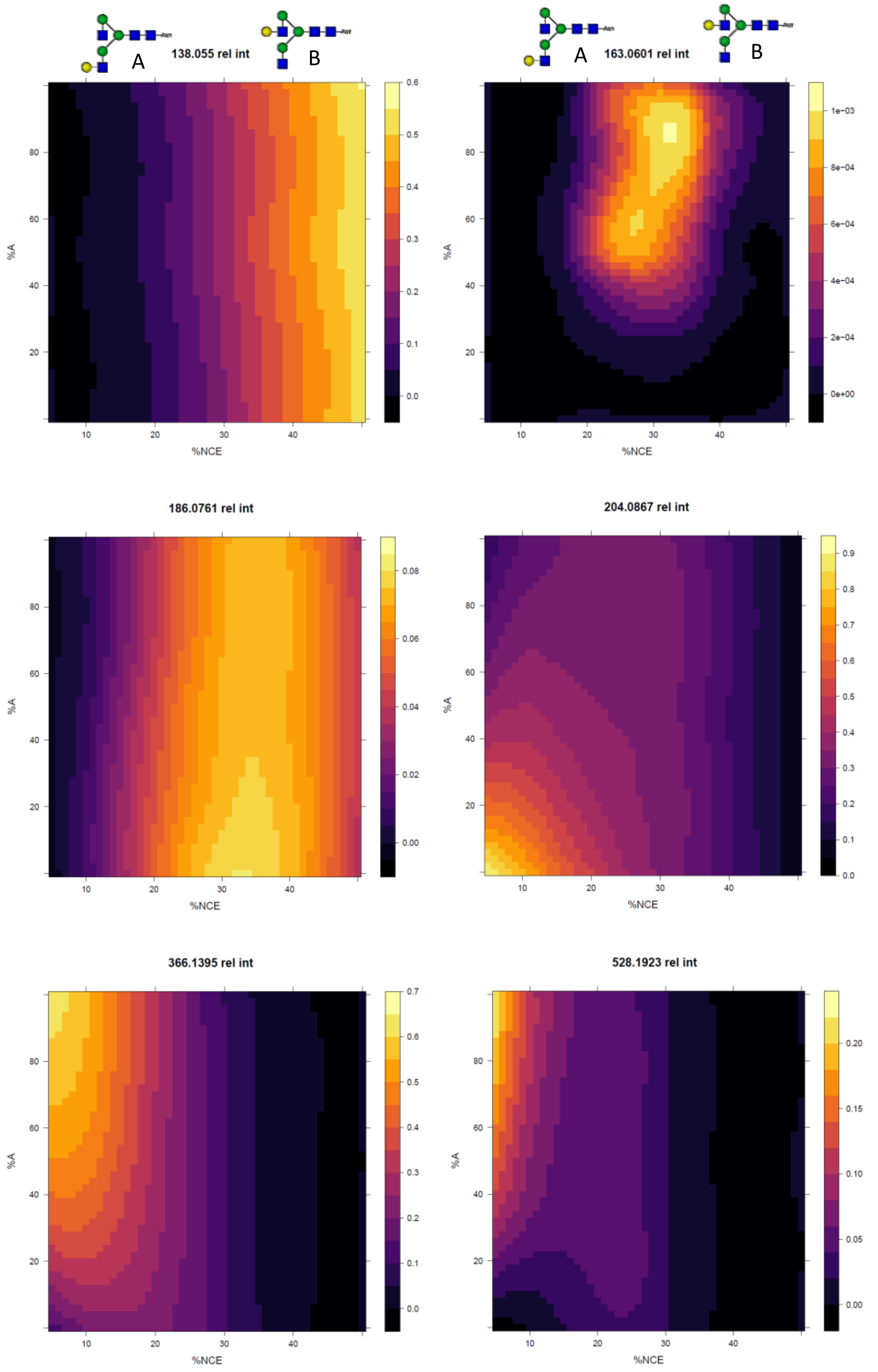

Figure S5. Relative intensities of oxonium ion for galactose branching. Relative intensities of oxonium ions are shown over a NCE range 5-50% and isomer mixtures ranging from 0-100% A. The color gradient indicates the relative intensity. HexNAc (*m/z* 204.0867) and HexHexNAc (*m/z* 366.1395) showed the biggest change between isomers.

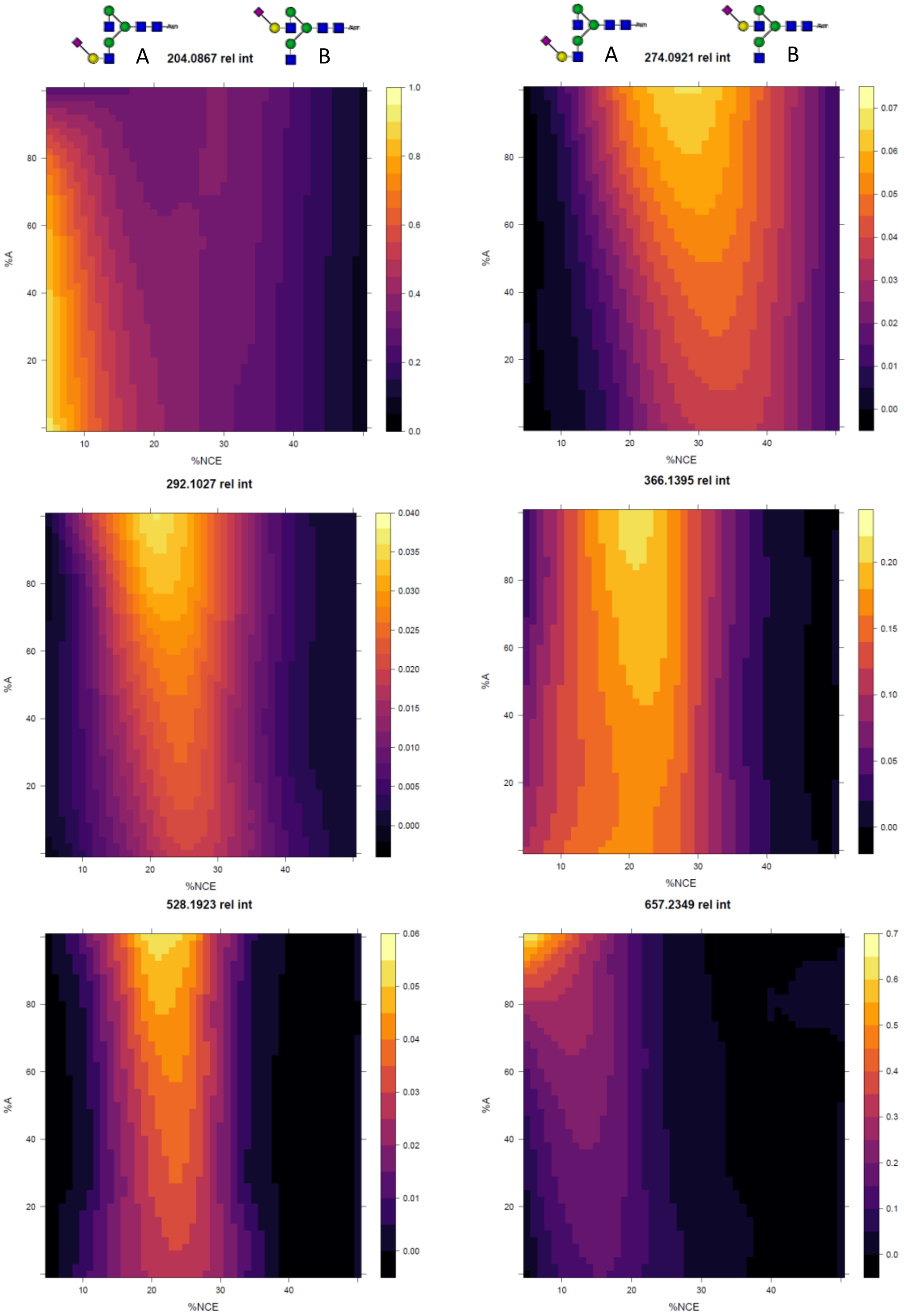

Figure S6. Relative intensities of oxonium ions for sialylated galactose branching. Relative intensities of oxonium ions are shown over a NCE range 5-50% and isomer mixtures ranging from 0-100% A. The color gradient indicates the relative intensity.

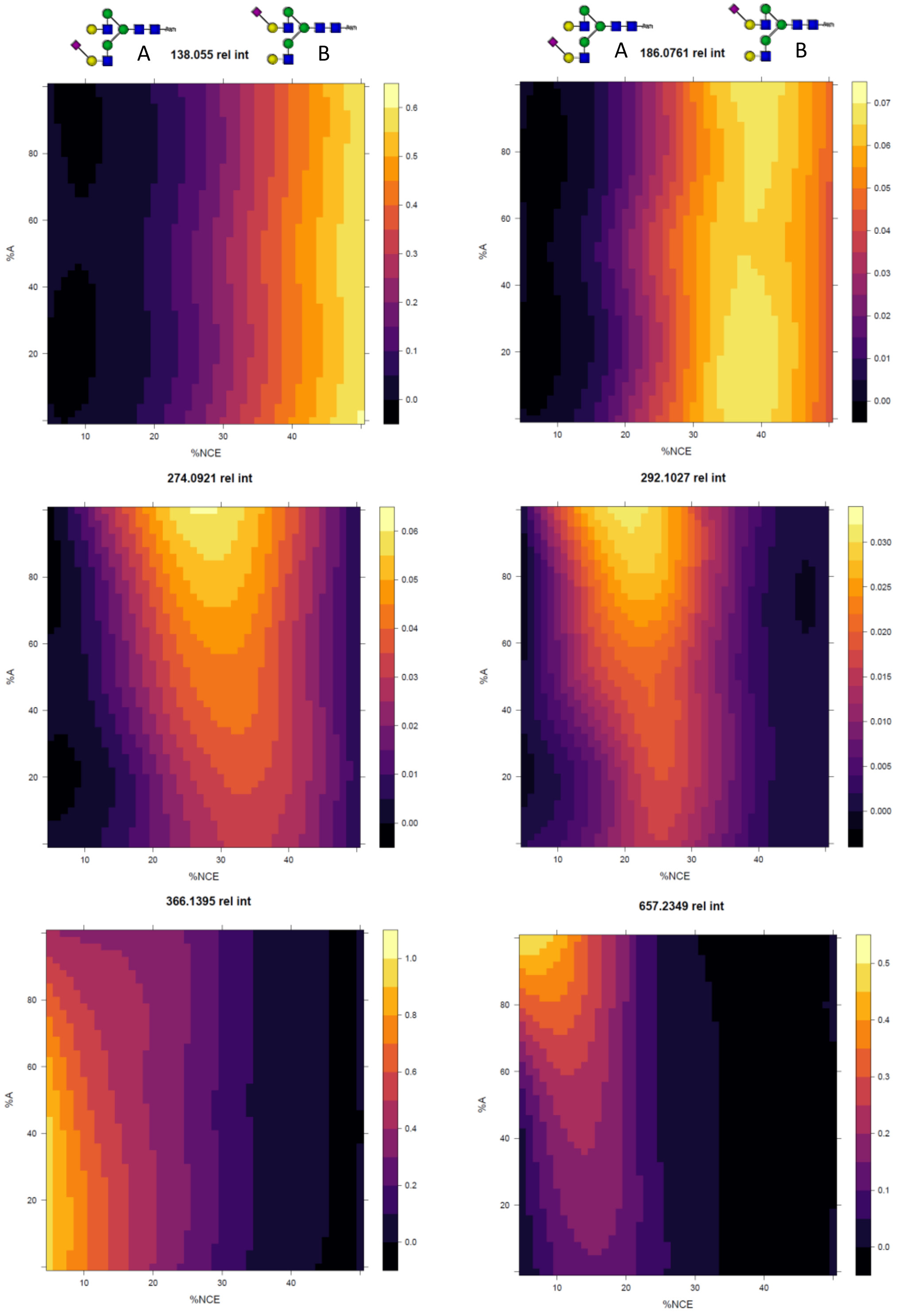

Figure S7. Relative intensities of oxonium ions for sialic acid branching. Relative intensities of oxonium ions are shown over a NCE range 5-50% and isomer mixtures ranging from 0-100% A. The color gradient indicates the relative intensity. HexHexNAc (*m/z* 366.1395) and NeuAcHexHexNAc (*m/z* 657.2349) showed the biggest change between isomers.

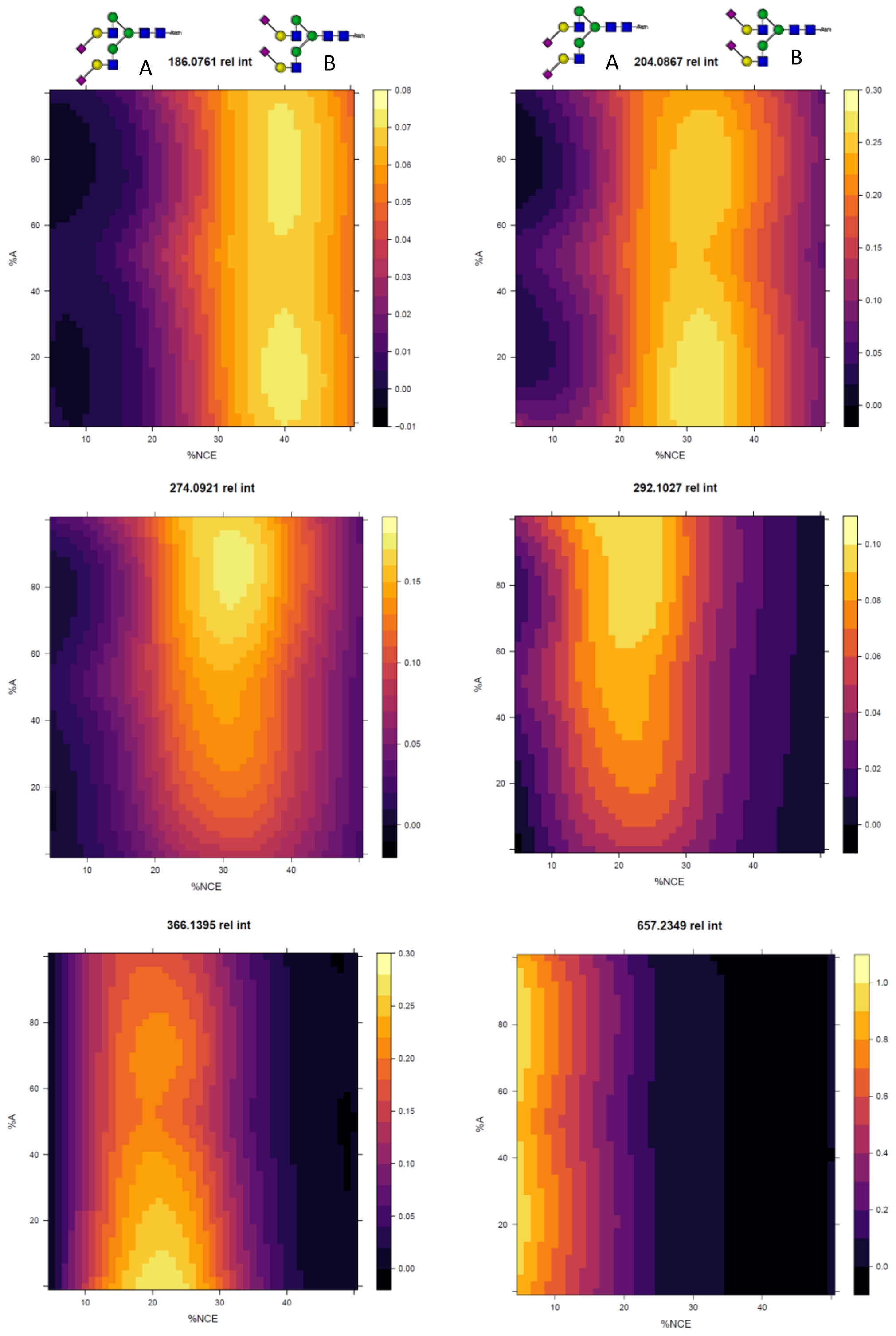

Figure S8. Relative intensities of oxonium ions for sialic acid linkage. Relative intensities of oxonium ions are shown over a NCE range 5-50% and isomer mixtures ranging from 0-100% A. The color gradient indicates the relative intensity. HexNAc (*m/z* 204.0867), HexHexNAc (*m/z* 366.1395), NeuAc (*m/z* 292.1027) and NeuAc-H2O (*m/z* 274.0921) showed the biggest change between isomers.

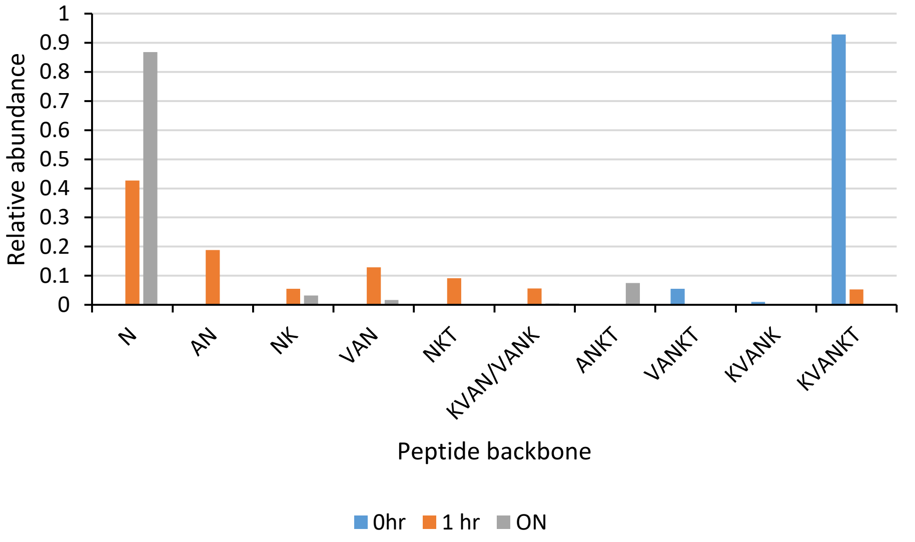

Figure S9: Partial proteolysis of SGP. SGP was digested with Pronase for 1 hour or overnight. After 1 hour, already over 40% of the SGP was fully digested, with only a single asparagine still being connected to the glycan. After overnight digestion almost 90% of the SGP was completely digested. The partial digestion of SGP created a range of glycopeptide with the same glycan but varying peptide backbones.

Table S1: Oxonium ion list for data analysis

Monosaccharide composition	m/z value
HexNAc - C2H6O3	126.055
Hex - 2H2O	127.039
HexNAc - CH6O3	138.055
HexNAc - C2H4O2	144.0655
Hex - H2O	145.0495
Hex	163.0601
HexNAc - 2H2O	168.0655
HexNAc - H2O	186.0761
HexNAc	204.0867
Hex + PO4	243.0264
NeuAc - H2O	274.0921
NeuGc - H2O	290.087
NeuAc	292.1027
NeuGc	308.0976
HexHexNAc	366.1395
Hex2 + PO4	405.0793
HexNAc2	407.166
NeuAcHex	454.1555
dHexHexHexNAc	512.1974
Hex2HexNAc	528.1923
HexHexNAc2	569.2188
NeuAcHexHexNAc	657.2349
Hex3HexNAc	690.2451
Hex2HexNAc2	731.2717
NeuAcHex2HexNAc	819.2877
Hex4HexNAc	852.2979
Hex3HexNAc2	893.3245
Hex2HexNAc3	934.351
NeuAcHex3HexNAc	981.3405
Hex4HexNAc2	1055.3773
Hex3HexNAc3	1096.4039
NeuAcHex4HexNAc	1143.3934
NeuAcHex3HexNAc2	1184.4199
Hex5HexNac2	1217.4301
Hex4HexNAc3	1258.4567
Hex3HexNAc4	1299.4832
NeuAcHex4HexNAc2	1346.4727
Hex5HexNAc3	1420.5095
Hex4HexNAc4	1461.5361
NeuAcHex5HexNAc2	1508.5256
NeuAcHex4HexNAc3	1549.5521
Hex6HexNAc3	1582.5623
Hex5HexNAc4	1623.5889
NeuAcHex5HexNAc3	1711.6049
Hex6HexNAc4	1785.6417
NeuAc2Hex5HexNAc2	1799.621
NeuAc2Hex5HexNAc3	2002.7003

Table S2. Overview of calculations to construct linkage variables for isomer determination.

Isomeric property	Oxonium ions used	%NCE used	Formula
Galactose branching	204.0867(HexNAc), 366.1395(HexHexNAc)	10,15,20,25	((rel int 204.0867@10%NCE/ rel int 204.0867@10%NCE + rel int 366.1395@10%NCE)
			+
			(rel int 204.0867@15%NCE/204.0867@15%NCE + rel int 366.1395@15%NCE)
			+
			(rel int 204.0867@20%NCE/ rel int 204.0867@20%NCE + rel int 366.1395@20%NCE)
			+
			(rel int 204.0867@25%NCE/ rel int 204.0867@25%NCE + rel int 366.1395@25%NCE))
			/4
Sialylated galactose branching	204.0867(HexNAc), 366.1395(HexHexNAc), 657.2349(HexHexNAcNeuAc)	10,15,20	((rel int 204.0867@10%NCE/ rel int 204.0867@10%NCE + rel int 366.1395@10%NCE + rel int 657.2349@10%NCE)
			+
			(rel int 204.0867@15%NCE/ rel int 204.0867@15%NCE + rel int 366.1395@15%NCE + rel int 657.2349@15%NCE)
			+
			(rel int 204.0867@20%NCE/ rel int 204.0867@20%NCE + rel int 366.1395@20%NCE + rel int 657.2349@20%NCE))
			/3
Sialic acid branching	366.1395(HexHexNAc), 657.2349(HexHexNAcNeuAc)	10,15	((rel int 366.1395@10%NCE/ rel int 366.1395@10%NCE + rel int 657.2349@10%NCE)
			+
			(rel int 366.1395@15%NCE/ rel int 366.1395@15%NCE + rel int 657.2349@15%NCE))
			/2
α2,3- and α2,6-sialylation	204.0867(HexNAc), 274.0921(NeuAc-H_2O), 292.1027(NeuAc), 366.1395(HexHexNAc)	20,25,30,35,40,45	((rel int 274.0921@20%NCE + rel int 292.1027@20%NCE/ rel int 274.0921@20%NCE + rel int 292.1027@20%NCE + rel int 204.0867@20%NCE + rel int 366.1395@20%NCE)
			+
			(rel int 274.0921@25%NCE + rel int 292.1027@25%NCE/ rel int 274.0921@25%NCE + rel int 292.1027@25%NCE + rel int 204.0867@25%NCE + rel int 366.1395@25%NCE)
			+
			(rel int 274.0921@30%NCE + rel int 292.1027@30%NCE/ rel int 274.0921@30%NCE + rel int 292.1027@30%NCE + rel int 204.0867@30%NCE + rel int 366.1395@30%NCE)
			+
			(rel int 274.0921@35%NCE + rel int 292.1027@35%NCE/ rel int 274.0921@35%NCE + rel int 292.1027@35%NCE + rel int 204.0867@35%NCE + rel int 366.1395@35%NCE)
			+
			(rel int 274.0921@40%NCE + rel int 292.1027@40%NCE/ rel int 274.0921@40%NCE + rel int 292.1027@40%NCE + rel int 204.0867@40%NCE + rel int 366.1395@40%NCE)
			+
			(rel int 274.0921@45%NCE + rel int 292.1027@45%NCE/ rel int 274.0921@45%NCE + rel int 292.1027@45%NCE + rel int 204.0867@45%NCE + rel int 366.1395@45%NCE))
			/6

Table S3. Overview of p-values obtained when performing a student's t-test between 10% isomer mixture steps.

Gal branching			NeuAcGal branching	
**Step**	**p-value**		**Step**	**p-value**
0-10	1.37E-06		0-10	3.55E-03
10-20	1.19E-13		10-20	1.09E-01
20-30	7.39E-05		20-30	1.99E-01
30-40	1.99E-06		30-40	9.86E-04
40-50	9.70E-08		40-50	6.95E-02
50-60	9.77E-03		50-60	2.97E-04
60-70	6.05E-02		60-70	4.20E-04
70-80	4.64E-04		70-80	9.84E-04
80-90	4.75E-03		80-90	6.14E-05
90-100	6.71E-01		90-100	4.05E-09
**NeuAc branching**			**NeuAc linkage**	
**Step**	**p-value**		**Step**	**p-value**
0-10	3.52E-01		0-10	1.64E-07
10-20	1.69E-04		10-20	1.58E-02
20-30	9.17E-01		20-30	4.80E-02
30-40	1.28E-02		30-40	1.71E-01
40-50	7.98E-01		40-50	1.67E-02
50-60	1.06E-02		50-60	8.75E-01
60-70	3.25E-02		60-70	3.58E-01
70-80	1.61E-01		70-80	3.78E-01
80-90	1.19E-02		80-90	1.30E-01
90-100	8.33E-02		90-100	2.53E-02

Supplementary Information 1

```
#! /usr/bin/env python

############################################
### Glycopeptide ismer data analysis###

# Libraries
import os
import sys
import re
import pandas as pd
from time import perf_counter


### Functions ####
def _LVCombine(dfs,LVName,NCEList):
	'''Generate LV from combined dfs lines.'''

	LV = 0.0
	for NCE in NCEList:
		LVs = dfs[dfs.index == NCE][LVName]

		if LVs.isnull().any():
			LVs.iat[0] = 0.0

		try:
			LV += LVs.iat[0]
		except IndexError:
			LV += 0.0

	LV = LV / len(NCEList)

	return(LV)


### Main loop ###
def main():
	'''Main loop.'''

	### Timing
	t1 = perf_counter()
```

Supplementary Information 1

```
### Input list control
        removeList = ["Unnamed","mz="," delta"," int/basepeak",
                        "scan number","id"]
        cycleName = "NCE"


        ### NCE cycle and removelists
        NCEcycle_single = ["29","30","10","50","20","40",
                        "15","45","25","35","05"]
        NCEcycle = NCEcycle_single*3
        removeDictNCE = {}
        for i in NCEcycle:
                removeDictNCE[i] = NCEcycle[:]
                removeDictNCE[i].remove(i)


        ### Ion m/z values - this could be read from file
        ionList = [ "126.055" , "127.039" , "138.055" , "144.0655",
                        "145.0495", "163.0601", "168.0655", "186.0761",
                        "204.0867", "243.0264", "274.0921", "290.087" ,
                        "292.1027", "308.0976", "366.1395", "405.0793",
                        "407.166" , "454.1555", "512.1974", "528.1923",
                        "569.2188", "657.2349", "690.2451", "731.2717",
                        "819.2877", "852.2979", "893.3245", "934.351" ,
                        "981.3405","1055.3773","1096.4039","1143.3934",
                "1184.4199","1217.4301","1258.4567","1299.4832",
                "1346.4727","1420.5095","1461.5361","1508.5256",
                "1549.5521","1582.5623","1623.5889","1711.6049",
                "1785.6417","1799.621" ,"2002.7003"]

        ionIList = []
        for i in ionList:
                ionIList.append(i + " int")


        ### Generating columns list from ion list
        columnList =["title","A","B","NCE","retention time",
                        "precursor mz","precursor int","precursor charge"]
        for i in ionList:
                columnList.append(i + " int")
        columnList = columnList + ["ion sum"]
        for i in ionList:
                columnList.append(i + " rel int")
```

Supplementary Information 1

```
columnList = columnList + ["int_time","linkage variable",
                                                     "sialic sum","hexnac sum",
                                                     "glycan check"]


        ### building block masses
        AADict = {"G": 57.02146,
                  "A": 71.03711,
                  "S": 87.03203,
                  "P": 97.05276,
                  "V": 99.06841,
                  "T":101.04768,
                  "C":103.00919,
                  "I":113.08406,
                  "L":113.08406,
                  "N":114.04293,
                  "D":115.02694,
                  "Q":128.05858,
                  "K":128.09496,
                  "E":129.04259,
                  "M":131.04049,
                  "H":137.05891,
                  "F":147.06841,
                  "R":156.10111,
                  "Y":163.06333,
                  "W":186.07931}

        ModDict = {"P": 79.96633,
                   "F":146.05791,
                   "H":162.05282,
                   "N":203.07937,
                   "S":291.09542,
                   "A":291.09542,
                   "G":307.09033,
                   "Carbamidomethylation": 57.02146,
                   "Water": 18.01057,
                   "Proton":1.00728,
                   "Electron":0.00055}


        ### Input potential glycopeptides
        gpList = ["EEQYNSTYR     N4H4",
                  "EEQYNSTYR     N4H4F1",
                  "EEQYNSTY      N4H4F1",
```

```
		"NSTY	N4H4F1",
			"EEQFNSTYR	N4H4",
			"EEQFNSTYR	N4H4F1",
			"EEQFNSTY	N4H4F1",
			"NSTY	N4H4F1",
			"EEQFNSTFR	N4H4",
			"EEQFNSTFR	N4H4F1",
			"EEQFNSTF	N4H4F1",
			"NSTF	N4H4F1",
			"EQYN	N4H4F1",
			"EQYN	N4H4",
			"YNST	N4H4F1",
			"YNST	N4H4",
			"EEQYN	N4H4F1",
			"EEQYN	N4H4",
			"EEQYNST	N4H4F1",
			"EEQYNST	N4H4",
			"EQYNST	N4H4F1",
			"EQYNST	N4H4",
			"TKPREEQYNSTYR	N4H4",
			"TKPREEQYNSTYR	N4H4F1",
			"TKPREEQYNSTY	N4H4F1",
			"TKPREEQFNSTYR	N4H4",
			"TKPREEQFNSTYR	N4H4F1",
			"TKPREEQFNSTY	N4H4F1",
			"TKPREEQFNSTFR	N4H4",
			"TKPREEQFNSTFR	N4H4F1",
			"TKPREEQFNSTF	N4H4F1",
			"LREEQFNSTFR	N4H4",
			"LREEQFNSTFR	N4H4F1",
			"LREEQFNSTF	N4H4F1",
			"TKLREEQFNSTFR	N4H4",
			"TKLREEQFNSTFR	N4H4F1",
			"TKLREEQFNSTF	N4H4F1",
			"TKPWEEQFNSTFR	N4H4",
			"TKPWEEQFNSTFR	N4H4F1",
			"TKPWEEQFNSTFR	N4H4F1"]

for i in range(len(gpList)):
	p,g = gpList[i].split()
	gpList[i] = p + " " + g


### Generating and storing glycopeptide masses
```

Supplementary Information 1

```
ppm = 10			# ppm tolerance for m/z values - was 5, maybe 10?
	ppm_MS1 = 10
	ppm_MS2 = 20
	zList = [2]			# In case of multiple charges

	gpDict = {}
	for gp in gpList:
		p,g = gp.split()

		for z in zList:
			zStr = str(z)
			zStrL = zStr + "_lower"
			zStrU = zStr + "_upper"
			gpDict[gp] = {"peptide":p,
							"glycan":g,
							zStr:0,
							zStrL:0,
							zStrU:0}

			# Calculate masses
			for aa in p:
				gpDict[gp][zStr] += AADict[aa]

			gList = re.split("(\d+)",g)
			for i in range(len(gList)):
				if gList[i].isdigit():
					continue
				elif not gList[i] == "":
					gpDict[gp][zStr] += (ModDict[gList[i]] *

float(gList[i+1]))

			gpDict[gp][zStr] += ModDict["Water"]
			gpDict[gp][zStr] += ModDict["Proton"]*z
			gpDict[gp][zStr] = round(gpDict[gp][zStr]/z,5)
			gpDict[gp][zStrL] = round(gpDict[gp][zStr] -
									(ppm / 1e6 *
gpDict[gp][zStr]),5)
			gpDict[gp][zStrU] = round(gpDict[gp][zStr] +
									(ppm / 1e6 *
gpDict[gp][zStr]),5)


	### Making list of files
	wd = "insertwdhere"
	if wd == "":
		wd = os.path.abspath("")
```

Supplementary Information 1

```
os.chdir(wd)

    fList = []
    for root, dirs, files in os.walk(".", topdown = False):
        for name in files:
            if ".txt" in name:
                fList.append(name)


    ### Reformatting and storing files within dataframe
    print("Reading files...")
    df = pd.DataFrame()
    for f in fList:
        print("...",f)
        d = pd.read_table(f,sep="\t",header=0)

        f = f.split("\\")[-1]
        f = f.split(".")[0]
        d["filename"] = f

        # Prevent issue when changing from 9.99 to 10.00 min RT
        d["rt_min"] = d["retention time"] / 60
        d["rt_minx10000"] = round(d["rt_min"]*1e4,0)
        d["rt_minx10000"] = d["rt_minx10000"].astype(str)
        d["rt_minx10000"] = d["rt_minx10000"].str.zfill(12)

        # Build and sort on unique rt+int values

        d["int_time"] = d["precursor int"].astype(str)+d["rt_minx10000"]
        d.sort_values("int_time",inplace=True)

        # Assign NCE values from NCEcycle
        #print("NCE cycle assignment...")
        cList = []
        int_prev = 0

        for int_data in d["precursor mz"]:
            if int_data == int_prev:
                cList.append(NCEcycle[NCEcycle.index(cList[-1])+1])
            else:
                cList.append(NCEcycle[0])
            int_prev = int_data

        d[cycleName] = cList[:] #recycle script change
```

Supplementary Information 1

```
# Calculate relative intensities
		#print("Relative intensity calculation...")
		d[ionIList]= d[ionIList].replace("-","0")
		d[ionIList]= d[ionIList].apply(pd.to_numeric,errors="coerce")
		d["ion sum"] = d[ionIList].sum(axis=1)
		for ion in ionList:
			d[ion + " rel int"] = d[ion + " int"] / d["ion sum"]
		# Peptide assignment
		#print("m/z-based glycopeptide assignment...")
		d["A"] = "-"
		d["Sequence"] = "-"
		d["Glycan"] = "-"
		for gp in gpDict:
			p,g = gp.split()
			for z in zList:
				gpzL = gpDict[gp][str(z)+"_lower"]
				gpzU = gpDict[gp][str(z)+"_upper"]

				d.loc[((d["precursor mz"] >= gpzL) &
					(d["precursor mz"] <= gpzU)),
					 "A"] = gp
				d.loc[((d["precursor mz"] >= gpzL) &
					(d["precursor mz"] <= gpzU)),
					 "sequence"] = p
				d.loc[((d["precursor mz"] >= gpzL) &
					(d["precursor mz"] <= gpzU)),
					 "glycan"] = g

		# Preliminary linkage variable calculations
		#print("Linkage variable calculations...")
		d["LV_3v6-branch Gal"]		=  (d["204.0867 rel int"] /

(d["204.0867 rel int"] +

d["366.1395 rel int"]))

		d["LV_3v6-branch SiaGal"]=  (d["204.0867 rel int"] /

(d["204.0867 rel int"] +

d["366.1395 rel int"] +

d["657.2349 rel int"]))

		d["LV_3v6-branch Sia"]		=  (d["366.1395 rel int"] /

(d["366.1395 rel int"] +

d["657.2349 rel int"]))
```

Supplementary Information 1

```
d["LV_3v6-linked Sia"]                    = ((d["274.0921 rel int"] +

            d["292.1027 rel int"])/

(d["274.0921 rel int"] +

d["292.1027 rel int"] +

d["204.0867 rel int"] +

d["366.1395 rel int"]))

                        # Add file to dataframe
                        df = df.append(d)


            ### Data quality control
            print("Data quality control...")
            ## Intensity threshold QC
            IntQC_204 = 1e4
            IntQC_274 = 5e3
            IntQC_292 = 5e3
            IntQC_366 = 1e4
            IntQC_657 = 1e4

            # 204
            df.loc[((df["204.0867 int"] >= IntQC_204) &
                        (df["NCE"] == "30")),"QC_204"] = "pass"
            df.loc[((df["204.0867 int"] <  IntQC_204) &
                        (df["NCE"] == "30")),"QC_204"] = "fail"

            # 274
            df.loc[((df["274.0921 int"] >= IntQC_274) &
                        (df["NCE"] == "30")),"QC_274"] = "pass"
            df.loc[((df["274.0921 int"] <  IntQC_274) &
                        (df["NCE"] == "30")),"QC_274"] = "fail"

            # 292
            df.loc[((df["292.1027 int"] >= IntQC_292) &
                        (df["NCE"] == "30")),"QC_292"] = "pass"
            df.loc[((df["292.1027 int"] <  IntQC_292) &
                        (df["NCE"] == "30")),"QC_292"] = "fail"

            # 366
            df.loc[((df["366.1395 int"] >= IntQC_366) &
                        (df["NCE"] == "30")),"QC_366"] = "pass"
            df.loc[((df["366.1395 int"] <  IntQC_366) &
                        (df["NCE"] == "30")),"QC_366"] = "fail"
```

Supplementary Information 1

```
# 657
        df.loc[((df["657.2349 int"] >= IntQC_657) &
                        (df["NCE"] == "30")),"QC_657"] = "pass"
        df.loc[((df["657.2349 int"] <  IntQC_657) &
                        (df["NCE"] == "30")),"QC_657"] = "fail"

        # Fill in lists
        for QC in ["QC_204","QC_274","QC_292","QC_366","QC_657"]:
                df.loc[:,QC]=df.loc[:,QC].ffill()

        ## RT window QC
        rtQC_lower = 10.0          # Should be set per analyte - via file?
        rtQC_upper = 20.0          # Stored in gpDict?

        df["QC_rt"] = "fail"
        for gp in gpDict:
                df.loc[((df["A"] == gp) &
                            (df["rt_min"] >= rtQC_lower) &
                            (df["rt_min"] <= rtQC_upper)),
                             "QC_rt"] = "pass"

        ### Removing and reorganizing columns
        print("Removing unnecessary columns...")
        # Removal
        for key in df.keys():
                for rm in removeList:
                        if rm in key:
                                df.drop(key, axis=1, inplace=True)


        ### Write to file
        print("Writing complete list to file...")
        print("... csv")
        df.set_index("NCE", inplace=True)
        df.to_csv(wd + "insertnamehere.csv")

        #print("... xlsx") # Slow compared to csv
        #writer = pd.ExcelWriter(wd + "\\_Combined.xlsx",
        #                                                    engine = "xlsxwriter",
        #
        options={"strings_to_numbers": True})
        #df.to_excel(writer, sheet_name = "all data")
        #writer.save()
        #writer.close()
```

```
        """
            ### Timing report
        t2 = perf_counter()
        print("Done! Elapsed time:",round(t2-t1,4),"seconds.")
        return(0)
"""

        ### Generate new dataset with one peptide per row
        print("Calculating precursor-specific linkage variables...")
        dfu = pd.DataFrame(columns = df.columns) # df unique
        uniqueList = df["precursor int"].unique()
        for unique in uniqueList:

            dfs = df[df["precursor int"] == unique] # df sub
            ## Inherit properties from NCE 29 scan
            dfs_29 = dfs[dfs.index == "30"]

            ## Linkage variable combinations
            # 3v6-branch Gal          (10-25%)
            LVName  = "LV_3v6-branch Gal"
            NCEList = ["10","15","20","25"]
            dfs_29.loc["30",LVName] = _LVCombine(dfs,LVName,NCEList)

            # 3v6-branch SiaGal               (10-20%)
            LVName  = "LV_3v6-branch SiaGal"
            NCEList = ["10","15","20"]
            dfs_29.loc["30",LVName] = _LVCombine(dfs,LVName,NCEList)

            # 3v6-branch Sia          (10-15%)
            LVName  = "LV_3v6-branch Sia"
            NCEList = ["10","15"]
            dfs_29.loc["30",LVName] = _LVCombine(dfs,LVName,NCEList)

            # 3v6-linked Sia          (20-45%)
            LVName  = "LV_3v6-linked Sia"
            NCEList = ["20","25","30","35","40","45"]
            dfs_29.loc["30",LVName] = _LVCombine(dfs,LVName,NCEList)

            # Assign and build new dataframe
            dfu = dfu.append(dfs_29,ignore_index=False)


        ### Write new dataset to file
        print("Writing unique precursors to file...")
```

```
        print("Writing unique precursors to file...")
        print("... csv")
        dfu.to_csv(wd + "\\insertnamehere.csv")


        ### Timing report
        t2 = perf_counter()
        print("Done! Elapsed time:",round(t2-t1,4),"seconds.")

        return(0)


if __name__ == "__main__":
        main()
        sys.exit()
```

Supplementary Information 2

```
### Installs ###
#install.packages("plot3D")
#install.packages("lattice")
#install.packages("viridis")
#install.packages("tidyverse")

### Imports ###
library(plot3D)
library(lattice)
library(viridis)
library(tidyverse)

### Variables ###
fn_input = "Insertfilename.txt"###input file
wd = "insertworkingdirectory"###working directory
subset_start = 67### first column with oxonium ion relative intensities.
subset_end = 113### Last column with oxonium ion relative intensities.

### Init ###
setwd(wd)

dat = read.table(fn_input,sep=",",header=1,na.string=c(NA,""),fill=T, check.names=FALSE)


##############################################################
### Boxplots, scatterplots and levelplots of oxonium ions###
##############################################################

### Boxplots
list_z = names(dat)[subset_start:subset_end]
list_A = levels(factor(dat$X.A.))

pdf(file="nameoutputfile.pdf")
par(mfrow=c(3,4))
for (var_z in list_z){
  boxplot(dat[,var_z]~dat$NCE,na.rm=T,
        ylim=c(0,1),
        xlab="%NCE",ylab="RI",main=var_z)
  for (A in list_A){
    dat_sub = dat[which(dat$X.A.==A),]
    boxplot(dat_sub[,var_z]~dat_sub$NCE,na.rm=T,
```

```
 ylim=c(0,1),
          xlab="%NCE",ylab="RI",main=paste(var_z,A))
  }
}
dev.off()


### Scatterplots
list_z = names(dat)[subset_start:subset_end]
list_A = levels(factor(dat$X.A.))

pdf(file="nameoutputfile.pdf")
par(mfrow=c(3,4))
for (var_z in list_z){
  plot(dat[,var_z]~dat$NCE,na.rm=T,
        ylim=c(0,1),
        xlab="%NCE",ylab="RI",main=var_z)
  for (A in list_A){
    dat_sub = dat[which(dat$A==A),]
    plot(dat_sub[,var_z]~dat_sub$NCE,na.rm=T,
          ylim=c(0,1),
          xlab="%NCE",ylab="RI",main=paste(var_z,A))
  }
}
dev.off()


### Level plot
list_z = names(dat)[subset_start:subset_end]
pdf(file="nameoutputfile.pdf")
for(var_z in list_z){
  df = data.frame(x=dat$NCE,y=dat$A,z=dat[,var_z])
  df.loess = loess(z~x*y,data=df,degree=2,span=0.25)
  df.fit = expand.grid(list(x = seq(5,50,1), y = seq(0,100,2)))
  z = predict(df.loess, newdata = df.fit)

  height = as.numeric(z)
  height[which(height<0)]=0

  print(height)

  z_plot = levelplot(height ~ x*y, data = df.fit,
                 xlab = "%NCE", ylab = "%A",
                 main = var_z,
```

```
 col.regions = inferno(100),
                   pretty = T
                   )
  print(z_plot)
}
dev.off()


######################################
### Linkage varaible calculations ###
######################################


###Linkage variable oxonium ion for galactose branching
#form_nom = c("204.0867 rel int")### nominator for linkage variable
#form_dnom = c("204.0867 rel int","366.1395 rel int")### denominator for linkage variable

###Linkage variable oxonium ion for sialylated galactose branching
#form_nom = c("204.0867 rel int")### nominator for linkage variable
#form_dnom = c("204.0867 rel int","366.1395 rel int","657.2349 rel int")### denominator for
linkage variable

###Linkage variable oxonium ion for sialic acid  branching
#form_nom = c("366.1395 rel int")### nominator for linkage variable
#form_dnom = c("657.2349 rel int","366.1395 rel int")### denominator for linkage variable

###Linkage variable oxonium ions for sialic acid linkage
form_nom = c("292.1027 rel int", "274.0921 rel int")### nominator for linkage variable
form_dnom = c("292.1027 rel int","366.1395 rel int","274.0921 rel int","204.0867 rel int")###
denominator for linkage variable


dat_NCE5 = dat[which(dat$NCE==5),]
dat_NCE10 = dat[which(dat$NCE==10),]
dat_NCE15 = dat[which(dat$NCE==15),]
dat_NCE20 = dat[which(dat$NCE==20),]
dat_NCE25 = dat[which(dat$NCE==25),]
dat_NCE30 = dat[which(dat$NCE==30),]
dat_NCE35 = dat[which(dat$NCE==35),]
dat_NCE40 = dat[which(dat$NCE==40),]
dat_NCE45 = dat[which(dat$NCE==45),]
dat_NCE50 = dat[which(dat$NCE==50),]

ms2_list = levels(factor(dat$"precursor int"))
```

Supplementary Information 2

```
ms2 = matrix(ms2_list)
ms2$int = ms2_list
ms2$A = array(dim=length(ms2_list))
ms2$NCE5 = array(dim=length(ms2_list))
ms2$NCE10 = array(dim=length(ms2_list))
ms2$NCE15 = array(dim=length(ms2_list))
ms2$NCE20 = array(dim=length(ms2_list))
ms2$NCE25 = array(dim=length(ms2_list))
ms2$NCE30 = array(dim=length(ms2_list))
ms2$NCE35 = array(dim=length(ms2_list))
ms2$NCE40 = array(dim=length(ms2_list))
ms2$NCE45 = array(dim=length(ms2_list))
ms2$NCE50 = array(dim=length(ms2_list))

for (int in ms2$int){
  ms2$A[which(ms2$int == int)] = dat$"A"[which(dat$"precursor int" == int)][1]
}

for (int in ms2$int){
  nom = dnom = 0
  for (num in form_nom){nom = nom + dat_NCE5[which(dat_NCE5$"precursor int" ==
int),num]}
  for (num in form_dnom){dnom = dnom + dat_NCE5[which(dat_NCE5$"precursor int"
== int),num]}
  if (length(nom/dnom) > 0){ms2$NCE5[which(ms2$int == int)] = nom/dnom}
}
for (int in ms2$int){
  nom = dnom = 0
  for (num in form_nom){nom = nom + dat_NCE10[which(dat_NCE10$"precursor int" ==
int),num]}
  for (num in form_dnom){dnom = dnom + dat_NCE10[which(dat_NCE10$"precursor
int" == int),num]}
  if (length(nom/dnom) > 0){ms2$NCE10[which(ms2$int == int)] = nom/dnom}
}
for (int in ms2$int){
  nom = dnom = 0
  for (num in form_nom){nom = nom + dat_NCE15[which(dat_NCE15$"precursor int" ==
int),num]}
  for (num in form_dnom){dnom = dnom + dat_NCE15[which(dat_NCE15$"precursor
int" == int),num]}
  if (length(nom/dnom) > 0){ms2$NCE15[which(ms2$int == int)] = nom/dnom}
}
for (int in ms2$int){
  nom = dnom = 0
  for (num in form_nom){nom = nom + dat_NCE20[which(dat_NCE20$"precursor int" ==
int),num]}
  for (num in form_dnom){dnom = dnom + dat_NCE20[which(dat_NCE20$"precursor
int" == int),num]}
  if (length(nom/dnom) > 0){ms2$NCE20[which(ms2$int == int)] = nom/dnom}
}
for (int in ms2$int){
  nom = dnom = 0
```

```
 for (num in form_nom){nom = nom + dat_NCE25[which(dat_NCE25$"precursor int" ==
int),num]}
  for (num in form_dnom){dnom = dnom + dat_NCE25[which(dat_NCE25$"precursor int" ==
int),num]}
  if (length(nom/dnom) > 0){ms2$NCE25[which(ms2$int == int)] = nom/dnom}
}
for (int in ms2$int){
  nom = dnom = 0
  for (num in form_nom){nom = nom + dat_NCE30[which(dat_NCE30$"precursor int" ==
int),num]}
  for (num in form_dnom){dnom = dnom + dat_NCE30[which(dat_NCE30$"precursor int" ==
int),num]}
  if (length(nom/dnom) > 0){ms2$NCE30[which(ms2$int == int)] = nom/dnom}
}
for (int in ms2$int){
  nom = dnom = 0
  for (num in form_nom){nom = nom + dat_NCE35[which(dat_NCE35$"precursor int" ==
int),num]}
  for (num in form_dnom){dnom = dnom + dat_NCE35[which(dat_NCE35$"precursor int" ==
int),num]}
  if (length(nom/dnom) > 0){ms2$NCE35[which(ms2$int == int)] = nom/dnom}
}
for (int in ms2$int){
  nom = dnom = 0
  for (num in form_nom){nom = nom + dat_NCE40[which(dat_NCE40$"precursor int" ==
int),num]}
  for (num in form_dnom){dnom = dnom + dat_NCE40[which(dat_NCE40$"precursor int" ==
int),num]}
  if (length(nom/dnom) > 0){ms2$NCE40[which(ms2$int == int)] = nom/dnom}
}
for (int in ms2$int){
  nom = dnom = 0
  for (num in form_nom){nom = nom + dat_NCE45[which(dat_NCE45$"precursor int" ==
int),num]}
  for (num in form_dnom){dnom = dnom + dat_NCE45[which(dat_NCE45$"precursor int" ==
int),num]}
  if (length(nom/dnom) > 0){ms2$NCE45[which(ms2$int == int)] = nom/dnom}
}
for (int in ms2$int){
  nom = dnom = 0
  for (num in form_nom){nom = nom + dat_NCE50[which(dat_NCE50$"precursor int" ==
int),num]}
  for (num in form_dnom){dnom = dnom + dat_NCE50[which(dat_NCE50$"precursor int" ==
int),num]}
  if (length(nom/dnom) > 0){ms2$NCE50[which(ms2$int == int)] = nom/dnom}
}
###replace NA values with 0
ms2$NCE5 = replace(ms2$NCE5,is.na(ms2$NCE5),0)
ms2$NCE10 = replace(ms2$NCE10,is.na(ms2$NCE10),0)
ms2$NCE15 = replace(ms2$NCE15,is.na(ms2$NCE15),0)
ms2$NCE20 = replace(ms2$NCE20,is.na(ms2$NCE20),0)
ms2$NCE25 = replace(ms2$NCE25,is.na(ms2$NCE25),0)
ms2$NCE30 = replace(ms2$NCE30,is.na(ms2$NCE30),0)
ms2$NCE35 = replace(ms2$NCE35,is.na(ms2$NCE35),0)
ms2$NCE40 = replace(ms2$NCE40,is.na(ms2$NCE40),0)
ms2$NCE45 = replace(ms2$NCE45,is.na(ms2$NCE45),0)
```

Supplementary Information 2

```
ms2$NCE50 = replace(ms2$NCE50,is.na(ms2$NCE50),0)

###Linkage variable NCE range for galactose branching
#ms2$NCEsum = (ms2$NCE10 + ms2$NCE15 + ms2$NCE20 + ms2$NCE25)/4

###Linkage variable NCE range for sialylated galactose branching
#ms2$NCEsum = (ms2$NCE10 + ms2$NCE15 + ms2$NCE20)/3

###Linkage variable NCE range for sialic acid branching
#ms2$NCEsum = (ms2$NCE10 + ms2$NCE15)/2

###Linkage variable NCE range for sialic acid linkage
ms2$NCEsum = (ms2$NCE20 + ms2$NCE25 + ms2$NCE30 + ms2$NCE35 + ms2$NCE40 +
ms2$NCE45)/6


### Place NA values back
ms2$NCEsum = replace(ms2$NCEsum,ms2$NCEsum <= 0,NA)
ms2$NCE5 = replace(ms2$NCE5,ms2$NCE5 <= 0,NA)
ms2$NCE10 = replace(ms2$NCE10,ms2$NCE10 <= 0,NA)
ms2$NCE15 = replace(ms2$NCE15,ms2$NCE15 <= 0,NA)
ms2$NCE20 = replace(ms2$NCE20,ms2$NCE20 <= 0,NA)
ms2$NCE25 = replace(ms2$NCE25,ms2$NCE25 <= 0,NA)
ms2$NCE30 = replace(ms2$NCE30,ms2$NCE30 <= 0,NA)
ms2$NCE35 = replace(ms2$NCE35,ms2$NCE35 <= 0,NA)
ms2$NCE40 = replace(ms2$NCE40,ms2$NCE40 <= 0,NA)
ms2$NCE45 = replace(ms2$NCE45,ms2$NCE45 <= 0,NA)
ms2$NCE50 = replace(ms2$NCE50,ms2$NCE50 <= 0,NA)

##################################
### Plotting linkage variable###
##################################

x = ms2$NCEsum
y = ms2$A


model = lm(x~y)
boxplot(x~y,xlab="%A",ylab="Linkage variable", ylim=c(0,1))
#abline(model)
#summary(model)

###############
### t.tests ###
###############
```

Supplementary Information 2

```
set1 = ms2$NCEsum[which(ms2$A == "0")]
set2 = ms2$NCEsum[which(ms2$A == "10")]
t.test(set1,set2)

set1 = ms2$NCEsum[which(ms2$A == "10")]
set2 = ms2$NCEsum[which(ms2$A == "20")]
t.test(set1,set2)

set1 = ms2$NCEsum[which(ms2$A == "20")]
set2 = ms2$NCEsum[which(ms2$A == "30")]
t.test(set1,set2)

set1 = ms2$NCEsum[which(ms2$A == "30")]
set2 = ms2$NCEsum[which(ms2$A == "40")]
t.test(set1,set2)

set1 = ms2$NCEsum[which(ms2$A == "40")]
set2 = ms2$NCEsum[which(ms2$A == "50")]
t.test(set1,set2)

set1 = ms2$NCEsum[which(ms2$A == "50")]
set2 = ms2$NCEsum[which(ms2$A == "60")]
t.test(set1,set2)

set1 = ms2$NCEsum[which(ms2$A == "60")]
set2 = ms2$NCEsum[which(ms2$A == "70")]
t.test(set1,set2)

set1 = ms2$NCEsum[which(ms2$A == "70")]
set2 = ms2$NCEsum[which(ms2$A == "80")]
t.test(set1,set2)

set1 = ms2$NCEsum[which(ms2$A == "80")]
set2 = ms2$NCEsum[which(ms2$A == "90")]
t.test(set1,set2)

set1 = ms2$NCEsum[which(ms2$A == "90")]
set2 = ms2$NCEsum[which(ms2$A == "100")]
t.test(set1,set2)
```
